# Mapping the potential distribution of high artemisinin-yielding *Artemisia annua *L. (*Qinghao*) in China with a geographic information system

**DOI:** 10.1186/1749-8546-5-18

**Published:** 2010-05-17

**Authors:** Linfang Huang, Caixiang Xie, Baozhong Duan, Shilin Chen

**Affiliations:** 1 Institute of Medicinal Plant Development, Chinese Academy of Medical Sciences and Peking Union Medical College, Beijing 100193, China

## Abstract

**Background:**

*Artemisia annua *L. is an important source for artemisinin, a potent drug for treating malaria. This study aims to map and predict the potential geographic distribution of *A. annua *L. in China.

**Methods:**

The Geographic Information System for traditional Chinese medicine (TCM-GIS) was developed and used to map the potential geographic distribution of *A. annua *L.

**Results:**

Climatic, edaphic and topographic characteristics of *A. annua *L. microhabitats in Youyang County were mapped to find distribution patterns. The maps identified that certain habitats in the Chongqing region and some potential regions, especially in Guizhou Province, possess similarity indices of ≥98%. In particular, high quality microhabitats *A. annua *L. were found in the Wuling mountains region.

**Conclusion:**

The present study demonstrates a GIS approach to predict potential habitats for *A. annua *L. TCM-GIS is a powerful tool for assessing bioclimatic suitability for medicinal plants.

## Background

*Artemisia annua *L. (*Qinghao*, Annual Wormwood) is a strongly fragrant, annual herbaceous plant used in Chinese medicine [1]. *A. annua *L. is the only natural botanical source for artemisinin (*Qinghaosu*) [2,3] and a potential source for essential oils for the perfume industry [4]. *A. annua *L. is now cultivated in China, Vietnam, India, Romania, Kenya and Tanzania [5]. Artemisinin, an endoperoxide sesquiterpene lactone in the aerial parts of *A. annua *L., is more efficacious, faster and less toxic than chloroquine in treating malaria. In addition, artemisinin is a potent anti-cancer agent, a possible antibacterial agent as well as a natural pesticide [6,7]. Chemical and biological synthesis of artemisinin is still under development due to poor yields [8-11]. Therefore, wild or cultivated *A. annua *L. is a major source for artemisinin [2,3,12].

The artemisinin content is highly dependent on plant ecotypes, ecological interactions, seasonal and geographic variations [13-18]. In fact, artemisinin is absent in some *A. annua *L. Artemisinin was first isolated in China and some Chinese germplasm has relatively higher artemisinin levels than those of Europe, North America, East Africa and Australia [2,13,16,17,19,20]. In Youyang County, Chongqing, China, the hometown of *A. annua *L., the plants have high (0.9%) levels of artemisinin. In 2006 the county became a national protected geographic area recognized by the General Administration of Quality Supervision, Inspection and Quarantine of China [21]. As the demand for artemisinin remains high around the world, finding suitable geographic regions for *A. annua *L. is a critical research area for the World Health Organization [22].

The geographic information system (GIS) technology manages geographic information with applications for various fields such as natural resources, transportation planning, environmental studies and vegetation distribution studies [23-26]. Recently updated, the geographic information system for traditional Chinese medicine (TCM-GIS) captures, stores, analyzes and displays geographically referenced information to analyze genetic, ecological and geographic patterns of the spatial distribution of a target species. Using the TCM-GIS, our previous studies analyzed the potential habitats and distributions of Chinese medicinal plants such as *Glycyrrhiza uralensis *Fisch., *Panax quinquefolium *and *Panax ginseng *[27-29]. The present study aims to characterize the eco-environmental conditions in the *A. annua *L. production areas in Youyang County and predict the potential distributions of *A. annua *L. with a high artemisinin-yielding potentials.

## Methods

### Data collection

The spatial distribution of *A. annua *L. was based on the following four sources: (1) the flora of China [30]; (2) scientific literature concerning the geographic distribution of *A. annua *L. in China [31]; (3) the Chinese Virtual Herbarium (CVH) [32], (4) germplasm accessions from the Sharing Information System for Chinese Medicinal Plant Germplasm Resources [33]; (5) field data of wild *A. annua *L. and interviews in Youyang County in 2008. Due to the excellent quality of *A. annua *L. from the habitats in Youyang County [31,34-36], a total of 180 accessions of *A. annua *L. germplasm were collected and used in the present study.

The potential distribution mapping program TCM-GIS and geo-referenced datasets were used to develop eco-adaptation models. The TCM-GIS package included three databases, namely (1) a basic geographic information database including digital line graphics and a digital elevation model (scale: 1:1,000,000), (2) a soil database (scale: 1:4,000,000), (3) and a climate database (mean values between 1971 and 2000). All three databases were used for spatial analysis and model calibration.

**Figure 1:**
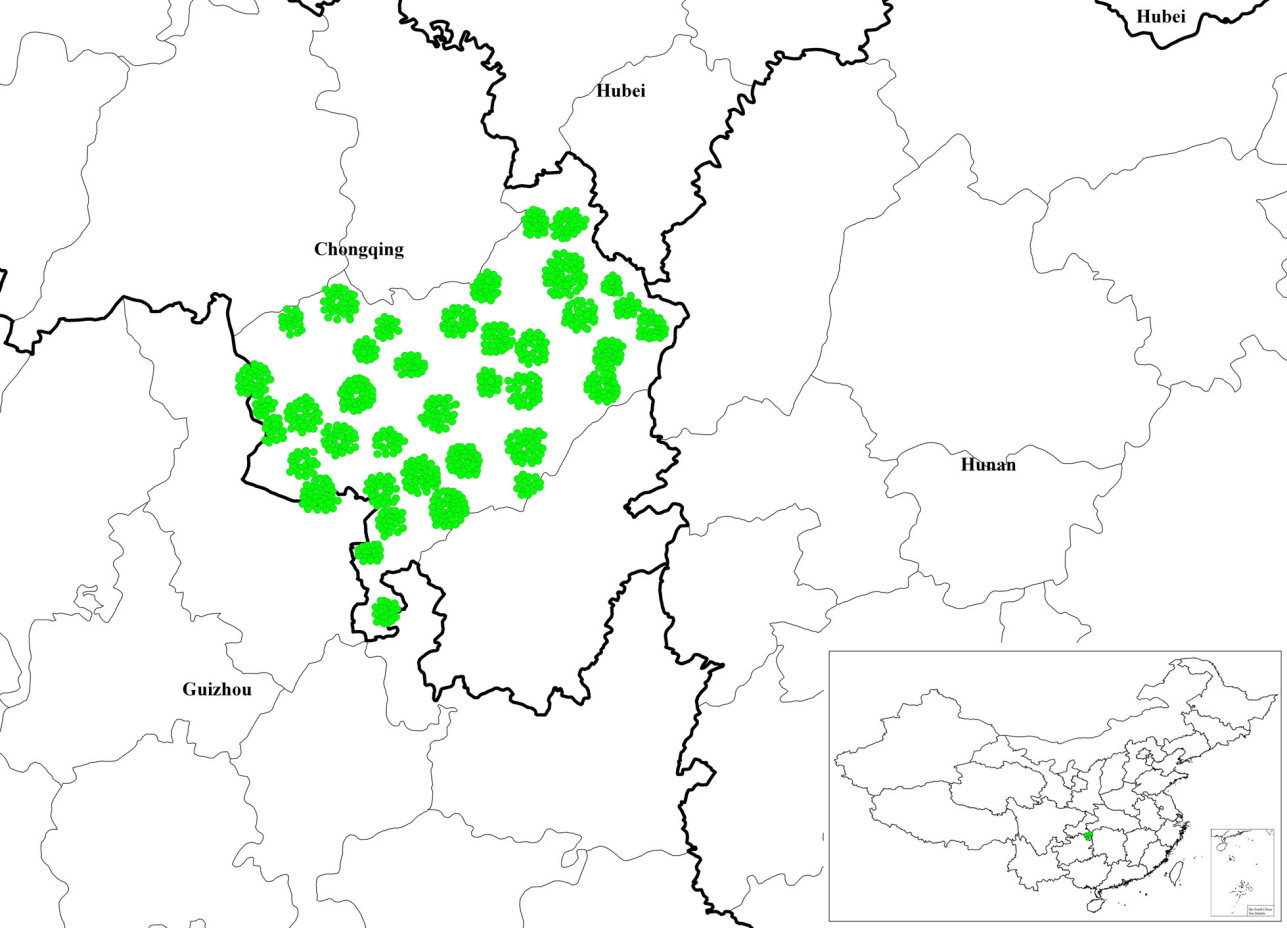
**Spatial distribution of *A. annua *L. germplasm collection sites as mapped with the TCM-GIS**.

Raster and vector are two main data models in the TCM-GIS. Raster layers (1 × 1 km^2 ^resolution) were used for the eco-environmental analysis and cluster analysis. Vector layers were used to derive and identify the spatial extent and location of suitable habitats through overlay analysis. Moreover, global positioning system data on the locations of the 180 accessions were obtained for villages such as Banqiao, Zhongduo, Mawang and Nanmu and used in the TCM-GIS analysis (Figure 1).

In the present study, 14 eco-environmental variables were chosen for the predication of spatial distribution in Youyang County. These variables, namely (1) average temperature in January (ATJA), (2) average temperature in February (ATF), (3) average temperature in March (ATM), (4) average temperature in April (ATAP), (5) average temperature in May (ATMA), (6) average temperature in June (ATJ), (7) average temperature in July (ATJU), (8) average temperature in August (ATA), (9) average annual temperature (AAT), (10) annual sunshine time (AST), (11) total annual precipitation (TAP), (12) relative humidity (RH), (13) altitude (AL), (14) and soil properties (SP), were classified into three categories: topography, climate and edaphology (Table 1).

**Table 1:** Environmental factors used in this study.

Category	Variables	Abbreviation
Climate	Average temperature in January (°C)	ATJA
	Average temperature in February (°C)	ATF
	Average temperature in March (°C)	ATM
	Average temperature in April (°C)	ATAP
	Average temperature in May (°C)	ATMA
	Average temperature in June (°C)	ATJ
	Average temperature in July (°C)	ATJU
	Average temperature in August (°C)	ATA
	Average Annual temperature (°C)	AAT
	Annual sunshine time (h)	AST
	Total annual precipitation (mm)	TAP
	Relative humidity (%)	RH
Topography	Altitude (m)	AL
Edaphology	Soil properties	SP

### Data analysis

An optimal range was established by identifying minima and maxima for eco-environmental variables (e.g. elevation and temperature) at sample collection sites. The *A. annua *L. macro-habitats were characterized by examining the mean, minimal and maximal values, standard deviation (SD), standard error (SE), and coefficient of variation (CV) of these variables (Table 2). Prior to distance analysis, we normalized the raster grid data representing each variable. We derived the mean absolute deviation using the following equation:

**Table 2:** Summary of eco-environmental characteristics from known *A. annua *L. habitats (*n *= 180).


**Variables**	**Mean**	**SE**	**CV%**	**SD**	**Range**	**Weight**

ATJA(°C)	3.95	0.005	21.46	0.849	1.2-5.6	0.03
ATF (°C)	4.1	0.005	21.43	0.765	2.0-6.0	0.03
ATM(°C)	8.50	0.005	13.36	1.136	4.0-10.0	0.06
ATAP(°C)	13.35	0.007	10.45	1.39	10.0-16.0	0.06
ATMA(°C)	17.92	0.006	6.81	1.22	14.0-20.0	0.08
ATJ(°C)	21.23	0.007	6.77	1.43	18.0-24.0	0.08
ATJU(°C)	25.30	0.06	4.60	1.164	21.6-27.3	0.08
ATA(°C)	23.56	0.08	6.23	1.469	20.0-26.0	0.08
AAT(°C)	19.32	0.05	4.69	0.907	15.9-21.0	0.08
AST(h)	1118.00	0.21	3.33	37.32	1048-1200	0.08
TAP(mm)	1209.00	0.09	1.28	15.46	1169-1267	0.08
RH(%)	79.85	0.02	0.33	2.63	79.2-80.6	0.15
AL(m)	771.03	1.28	29.79	229.73	498-1010	0.03
SP*						0.08

**Figure 2:**
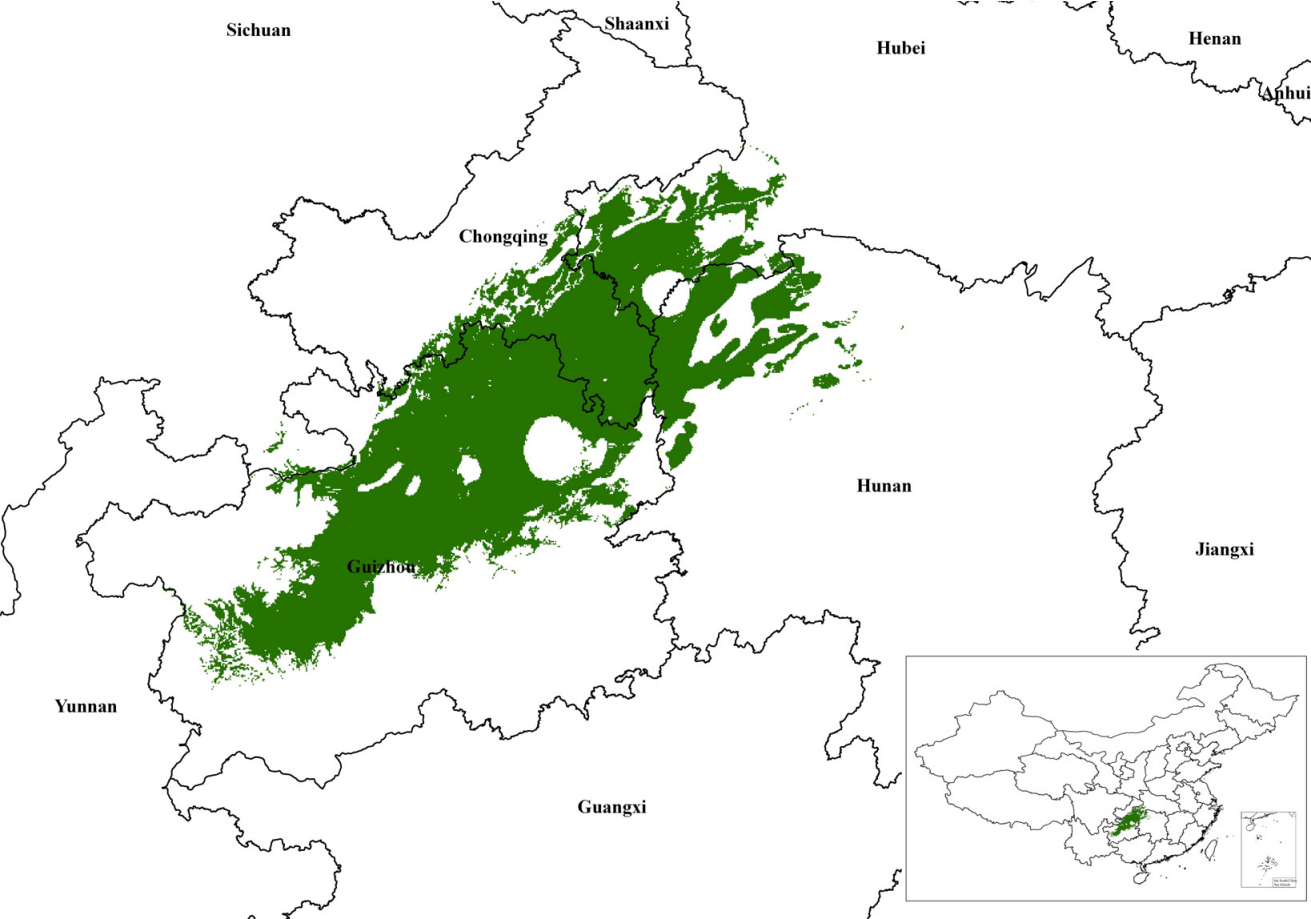
Distribution of suitable *A. annua *L. production areas in China with a similarity index (SI) of 99-100%

**Figure 3:**
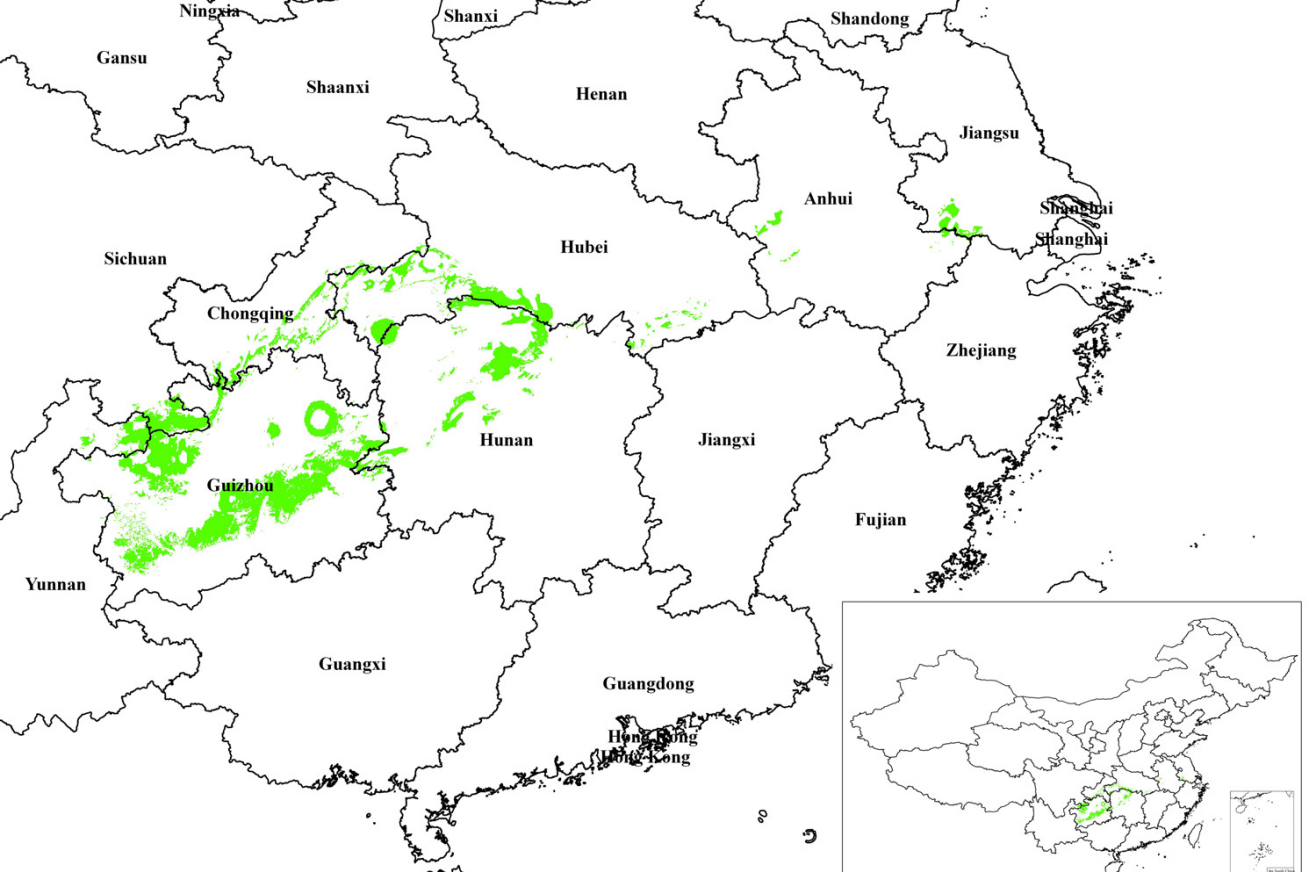
**Distribution of suitable *A. annua *L. production areas in China with a similarity index (SI) of 98-99%**.

**Figure 4:**
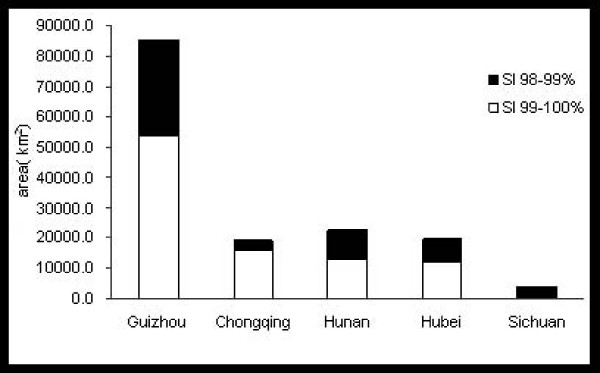
**Suitable regions for *A. annua *L. production with a similarity index (SI) of ≥98%**.

*Indication of five soil types: yellow soil, yellow sandy soil, limestone soil, paddy soil and brown soil; pH: 6-7; organic matter content ≥1.3%

where x_kf _was the measured values of the variable f and m_f _is the mean for the variable f. For the determination of similarity between grid data and eco-factor ranges, the statistical distance was calculated with the Minkowski distance equation [37]:

which is a generalization of the Euclidean distance and Manhattan distance; in general the shorter the distance, the greater the similarity. The comprehensive similarity index (SI) of each factor layer was calculated with an overlay analysis with various weighting values. Finally, maps with two ranks of predictive distributions were generated, followed by a grid-based spatial cluster analysis, vector-based overlaying, intersection analysis and an area calculation (Figures 2, 3, 4, Table 3).

**Table 3:** Major *A. annua *L. regions with similarity index (SI) of 99%-100%.

County/City, Province*	Suitable areas km^2^	Suitable areas %	County/City, Province	Suitable areas km^2^	Suitable areas %
Youyang, Chongqing	4386	92	Hefeng, Hubei	1225	46
Xiushan, Chongqing	1419	63	Enshi, Hubei	2038	55
Wulong, Chongqing	1290	48	Zunyi, Guizhou	3264	70
Qiangjiang, Chongqing	2286	97	Zhijin, Guizhou	1594	62
Pengshui, Chongqing	3182	87	Zhengan, Guizhou	1590	67
Zhangjiajie, Hunan	1388	58	Yanhe, Guizhou	1471	65
Yongshun, Hunan	1863	52	Xixiu, Guizhou	1387	90
Shangzhi, Hunan	1966	61	Wuchuan, Guizhou	2119	82
Longshan, Hunan	2017	69	Tongzi, Guizhou	1579	53
Baojing, Hunan	1235	77	Shuiyang, Guizhou	1463	62
Xuanen, Hubei	1909	74			
Xianfengshi, Hubei	2257	96			
Lichuan, Hubei	2266	52	Others are omitted		

*Areas smaller than 1400 km^2 ^are not listed.

The most favorable region for *A. annua *L. growth is one that has an SI range of 99%-100%, while the second-most favorable region is one that has an SI range of 98%-99%.

## Results and Discussion

### Eco-environmental preferences

The climatic, edaphic and topographic characteristics of known *A. annua *L. habitats are listed in Table 2. While low CV values for RH (CV: 0.33), TAP (1.28), AST (3.33), ATJU (4.60), AAT (4.69), ATA (6.23), ATJ (6.77) and ATMA (6.81) suggested that these could be the major limiting factors affecting the distribution of high quality *A. annua *L., high CV values for AL (29.79), ATJA (21.46) and ATF (21.43) suggested otherwise. According to the CV values, weighting value for each parameter was divided into levels I (0.15), II (0.08), III (0.06) and IV (0.03) and weighting values should add up to one. In addition, datasets of eco-factors from known habitats in Youyang County were as follows: ATJA = 1.2-5.6°C, ATF = 2.0-6.0°C, ATM = 4.0-10.0°C, ATAP = 10.0-16.0°C, ATMA = 14.0-20.0°C, ATJ = 18.0-24.0°C, ATJU = 21.6-27.3°C, ATA = 20.0-26.0°C, AAT = 15.9-21.0°C, AST = 1048-1200 h, TAP = 1169-1267 mm, RH = 79.2-80.6%, AL = 498-1010 mm. Soil types were mainly yellow soil, yellow sandy soil, limestone soil, paddy soil and brown soil with pH value at 6-7 and organic matter content ≥1.3%. Thus, we assumed that these conditions were optimal for the growth of high artemisinin-yielding *A. annua *L.

*A. annua *L. is a short-day plant. Non-juvenile plants are very responsive to short photoperiodic stimuli and flower about two weeks after induction. They require about 1000 hours of sunlight per year. Our results suggest that annual sunlight time is a critical factor for the growth of *A. annua *L., which is consistent with previous studies [5,38]. Previous findings that *A. annua *L. requires a strict watering regime during the preliminary growth stages [5,39] are also consistent with our results.

### Predictive maps

Figures 2 and 3 are the maps derived from the TCM-GIS analyses. The predicted areas were primarily located in the Wuling Mountain region in central China, covering Guizhou, Chongqing, Hunan, Hubei and Sichuan (25°14'-31°38' N to 104°31'-111°51'E). The predicted habitat density was high in northeastern Guizhou, southeastern Chongqing, northwestern Hunan, southwestern Hubei and parts of southern Sichuan.

The total favorable regions (SI 98%-99%) made up 1.60% of China's total land area covering 162 counties and cities (a total of 60,292 km^2^), among which Guizhou took the lead with 31,150 km^2 ^including 68 counties and cities. The most favorable region for *A. annua *L. (SI 99%-100%) was in the 58 counties and cities in Guizhou Province with a predicted area of 54,350 km^2^. The second largest predicted area (14,330 km^2^) was in the 12 counties and cities in Chongqing, followed by Hunan, Hubei and Sichuan (Figure 4). The counties and cities with significant areas of potential habitat are listed in Table 3. The data indicated that Youyang County contained the largest favorable area with more than 4000 km^2^. Unexpectedly, the total predicted areas in Wuchuan and Zunyi Counties in Guizhou exceeded 2000 km^2^.

One of the world's largest artemisinin manufacturers and its affiliates operate *A. annua *L. farms in the Chongqing Wulingshan Mountain Range [40,22]. Apart from this, Guizhou may be another important region for *A. annua *L. cultivation, particularly in the northeastern part of the province. Our model predicted that 13% of this area is potential *A. annua *L. habitats [41,42]. Our model did not predict Guangxi Province, known for its habitats of *A. annua *L. of relatively low quality, as a region for *A. annua *L. cultivation possibly due to the subtropical climate, low altitude and red soil in Guangxi which are very different from those in other *A. annua *L. regions in China [9].

Interviews with the locals suggest that the Guizhou region and Youyang County have comparative advantages for *A. annua *L. growth with a high-yield variety and minimal pests. Furthermore, the northeastern Guizhou is home to wild populations of *A. annua *L. which may be an alternative source for artemisinin.

Using the TCM-GIS, we aimed to determine the optimal ecological factors from known habitats and the results showed that RH, TAP, AST, STJU, AAT and SP were important limiting factors. We also aimed to map the distribution of potential regions for the development of *A. annua *L. in China based on selected climatic, soil and topographical values. Using bioclimatic similarity theory and the TCM-GIS, we predicted the potential growing areas at the county level, particularly in northeastern Guizhou Province. The TCM-GIS is adequate for predicting and identifying potential areas for *A. annua *L. cultivation.

Using a higher resolution raster and vector spatial databases, we improved the resolution of species distribution considerably on the national surveys conducted in the 1960s, 1970s and 1980s. While most of the survey data were based largely on personal experiences and rough estimates, the model used in the present study is relatively objective.

## Conclusion

The present study demonstrates a GIS approach to predict the potential habitats for *A. annua *L. TCM-GIS is a powerful tool for assessing bioclimatic suitability for medicinal plants.

## Abbreviations

TCM-GIS: traditional Chinese medicine geographic information system; GIS: geographic information system; SI: similarity index; SD: standard deviation; SE: standard error; CV: coefficient of variation; ATJA: average temperature in January; ATF: average temperature in February; ATM: average temperature in March; ATAP: average temperature in April; ATMA: average temperature in May; ATJ: average temperature in June; ATJU: average temperature in July; ATA: average temperature in August; AAT: average annual temperature; AST: annual sunshine time; TAP: total annual precipitation; RH: relative humidity; AL: altitude; SP: soil properties; CVH: Chinese Virtual Herbarium.

## Competing interests

The authors declare that they have no competing interests.

## Authors' contributions

LFH, SLC and CXX designed the study and performed the analyses. BZD helped with data analysis. All authors wrote the manuscript. All authors read and approved the final version of the manuscript.
